# Up-regulation of Plasma Hexosylceramide (d18:1/18:1) Contributes to Genotype 2 Virus Replication in Chronic Hepatitis C: A 20-Year Cohort Study

**DOI:** 10.1097/MD.0000000000003773

**Published:** 2016-06-10

**Authors:** Jin-Yan Zhang, Feng Qu, Jun-Feng Li, Mei Liu, Feng Ren, Jing-Yun Zhang, Dan-Dan Bian, Yu Chen, Zhong-Ping Duan, Jin-Lan Zhang, Su-Jun Zheng

**Affiliations:** From the Artificial Liver Center, Beijing YouAn Hospital, Capital Medical University, Beijing, China (J-YZ, ML, FR, J-YZ, D-DB, YC, Z-PD, S-JZ); State Key Laboratory of Bioactive Substance and Function of Natural Medicines, Institute of Materia Medica, Chinese Academy of Medica Sciences & Peking Union Medical College, Beijing, China (FQ, J-LZ); and Institute of Infectious Diseases, Department of Infectious Diseases, the First Hospital of Lanzhou University, Lanzhou, China (J-FL).

## Abstract

The aim of the present study was to explore the relationship between plasma sphingolipids and hepatitis C virus (HCV) replication in chronic hepatitis C (CHC) patients.

A cohort of 120 treatment-naïve CHC patients was included. Liver biopsies and the Scheuer scoring system were used to assess hepatic inflammatory activity. Blood biochemical indicators, HCV-RNA load, and immunological markers were also measured. Forty-four plasma sphingolipids were identified and quantified using high-performance liquid chromatography–tandem mass spectrometry.

The hexosylceramide (HexCer) (d18:1/18:1) level was significantly different between patients with a low HCV load (<10^6^ IU/mL) and a high HCV load (≥10^6^ IU/mL), and it was positively correlated with the HCV-RNA load (*r* = 0.337, *P* = 0.001) in CHC patients. Additionally, the plasma HexCer (d18:1/18:1) level (odds ratio 1.302, 95% confidence interval 1.129–1.502) was an independent factor for a high HCV-RNA load. For patients with hepatic inflammation grade ≤2 or HCV genotype 2, HexCer (d18:1/18:1) was independently related to a high HCV-RNA load.

Plasma HexCer (d18:1/18:1) might be involved in the high viral replication level in chronic HCV infection, especially for CHC patients with genotype 2.

## Abstract 2

Supplemental Digital Content is available in the text

## INTRODUCTION

Hepatitis C virus (HCV) infection has been recognized as a leading cause of chronic liver disease, since it was discovered in 1989.^1^ Approximately 3% of the population worldwide—130 to 170 million people—has an HCV infection.^2^ Approximately 10% to 20% of patients with chronic HCV infection progress to cirrhosis in a period of 20 to 30 years.^3^ Although many factors are associated with disease progression, persistent HCV replication has been recognized as one of the main determinants of hepatic deterioration.^4^ Suppression of viral replication is an important strategy for preventing the progression of chronic liver disease. Although the combination of pegylated interferon-α and ribavirin or direct-acting antiviral agents could remove the viruses, a few patients suffer from treatment failure, and they require treatment in the future. Therefore, further searches for novel targets that are a closely related to the HCV replication process are urgently needed.

Sphingolipids interact with cholesterol and glycosphingolipid during the formation of lipid rafts. These provide a platform for signal transduction and pathogen infection,^5^ and also play crucial roles in the viral life cycle.^6^ For instance, lipid rafts may be the sites for HCV-RNA synthesis.^7^ Recent studies have demonstrated that sphingolipids are closely related to liver disease, especially ceramide metabolites.^8^ Ceramides play an essential role in inhibiting HCV entry.^9^ During HCV infection, the suppression of sphingolipid biosynthesis with a serine palmitoyltransferase inhibitor, which is the first-step enzyme in the ceramide biosynthetic pathway, could suppress viral replication in HCV-infected chimeric mice and HCV replicon cells.^10–12^ Additionally, HCV infection regulates various aspects of lipid metabolism within infected hepatocytes.^13–15^ For example, HCV upregulates the sphingomyelin and ceramide levels, promoting viral replication in the hepatocytes of humanized chimeric mice.^16^ Although multiple reports have indicated that dysregulation of sphingolipid synthesis affects HCV replication in animal models and cells, the relationship of plasma sphingolipids with HCV replication in chronic hepatitis C (CHC) patients remains poorly understood.

Previously, we established mature high-performance liquid chromatography–tandem mass spectrometry (HPLC-MS/MS) techniques to quantify sphingolipids.^17,18^ On the basis of a platform that improved quantitative high-throughput lipidomic, we initially confirmed a relationship between the plasma sphingolipids and hepatic inflammation in CHC.^19^ Subsequently, we demonstrated that glycosphingolipid and sphingomyelin were closely related to hepatic steatosis and fibrosis in CHC patients, respectively.^20,21^ On the basis of the previous reports and studies, we hypothesized that one or a subset of sphingolipids might be involved in HCV replication.

Therefore, in the present study, with the help of HPLC-MS/MS, liver biopsy, and HCV-RNA quantification, we aimed to evaluate the relationship between plasma sphingolipids and HCV replication among treatment-naïve patients with CHC, and also the subgroup of patients without significant necroinflammation (grade ≤2) or with HCV genotype 2.

## METHODS

### Patients

A cohort of 122 treatment-naïve CHC patients with a clear history of paid plasma donation from 1992 to 1995, from Dingxi City (Gansu Province, China), who were followed between July 2010 to June 2011, were enrolled in the present study.^22^ The CHC diagnosis was made in accordance with established criteria.^23,24^ Patients were excluded if they were coinfected with HBV, human immunodeficiency virus (HIV), or hepatitis delta virus (HDV), or if they had a malignant disease, including hepatocellular carcinoma, or there were no biopsy results. Two cases were excluded; one was for ascites, and the other one was because the liver biopsy specimen volume was too small. One hundred twenty patients were eligible for the study. Informed consents were obtained. The study was performed according to the provisions of the Declaration of Helsinki and was approved by the Institutional Review Board of Beijing YouAn Hospital, Capital Medical University, Beijing, China.

### Clinical Date Collection

The blood biochemical indicators, HCV-RNA load, and immunological markers were collected as previously described.^22^ Fasting blood samples were collected at the time of biopsy and stored at −80°C. Routine serological indications were extracted in all patients.

### Liver Biopsy

Liver biopsies were performed as described.^22^ The Scheuer scoring system was used to assess hepatic inflammatory activity.^25^ We selected patients without significant necroinflammation (grade ≤2) for the subgroup statistical analysis.

### HPLC-MS/MS

Serum sphingolipids were detected by HPLC-MS/MS as previously described.^17^ Sphingolipidomic assays were performed at the Institute of Materia Medica, Peking Union Medical College (Beijing, China).

### Statistical Analysis

Data were expressed as mean ± standard deviation (SD), unless otherwise specified. Statistical comparisons for continuous variables were performed using the independent-samples *t* test or Mann–Whitney *U* test. Categorical variables were analyzed using the Pearson chi-square test. Correlation analysis between the plasma sphingolipids and HCV-RNA load was made using Spearman rank correlation. After univariate analysis, stepwise logistic regression analysis with a forward selection, and the *P* values of entry and removal were set to 0.05 and 0.1, respectively. Statistical analysis was performed using SPSS version 19.0 (Chicago, IL). A *P* value <0.05 was considered statistically significant.

## RESULTS

### Clinical Characteristics of Patients

In our cohort, there were 57 (47.5%) male and 63 (52.5%) female patients who had a mean age of 51.33 years. The mean serum alanine aminotransferase (ALT) and aspartate aminotransferase (AST) values were 60.42 and 47.94 U/L, respectively; they were mildly elevated at the time of biopsy. According to the Scheuer inflammation score, grade 2 was present in 67 patients, accounting for the largest proportion (55.8%). This was followed by grades 3 (28.3%, 34/120) and 1 (13.3%, 16/120). Grades 0 and 4 were found in 0.8% (1/120) and 1.7% (2/120) of the patients, respectively. The number of patients presenting with an HCV-RNA load less than 10^6^ IU/mL was 72 (60%); 48 (40%) patients had more than 10^6^ IU/mL. In terms of genotypes, 44.2% (53/120) of patients had HCV genotype 2, which was closely followed by HCV genotype 1 (36.7%, 44/120). The remainder did not belong to either genotype 1 or genotype 2 (19.2%) (Table 1).

**TABLE 1:**
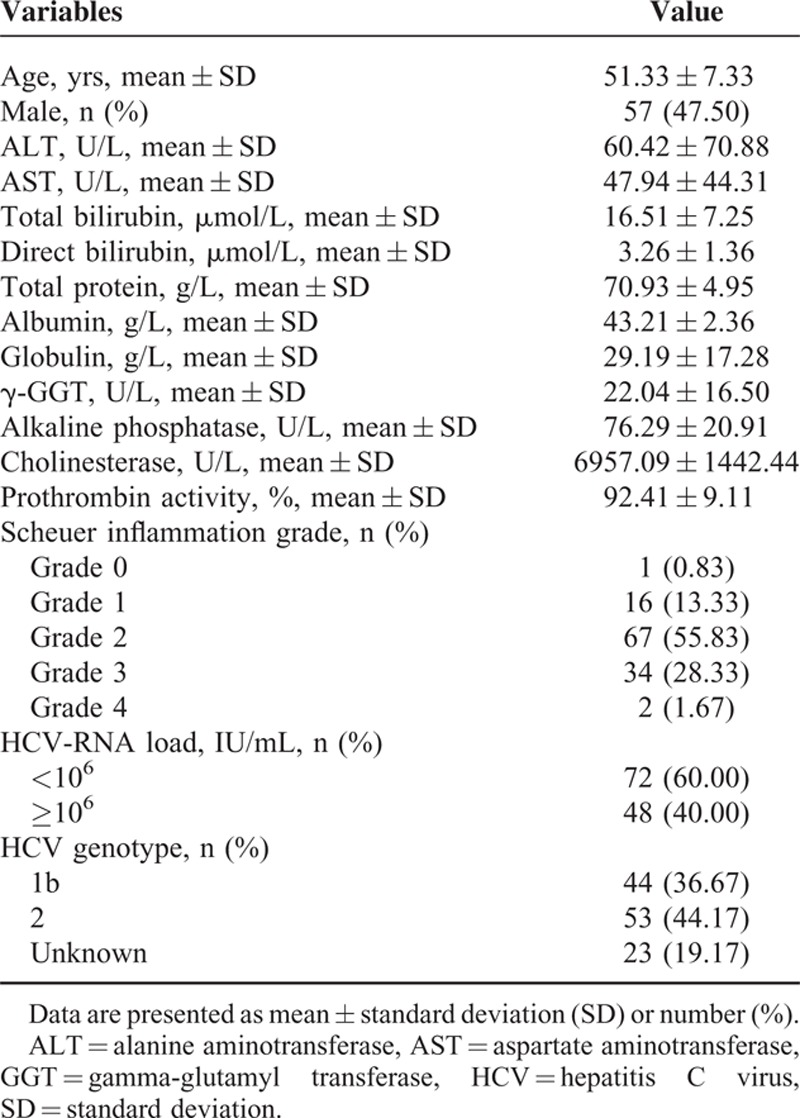
Demographic and Clinical Characteristics of the Patients With Chronic Hepatitis C (n = 120)

### Relationship Between Plasma Sphingolipids and HCV-RNA Load in CHC Patients

Plasma sphingolipids in the 120 patients were detected by HPLC-MS/MS. Forty-four sphingolipids were identified and quantified. Twenty-two plasma sphingolipids showed a significant difference between patients with a low viral load (LVL, HCV-RNA load **<**10^6^ IU/mL) and a high viral load (HVL, HCV-RNA load ≥10^6^ IU/mL) (*P* < 0.05) (Table 2). To further analyze the correlation between these differentially expressed sphingolipids and HCV-RNA load, Spearman correlation analysis was performed. The results of the correlation analysis revealed that 9 sphingolipids were correlated with the HCV-RNA load (Table 3).

**TABLE 2:**
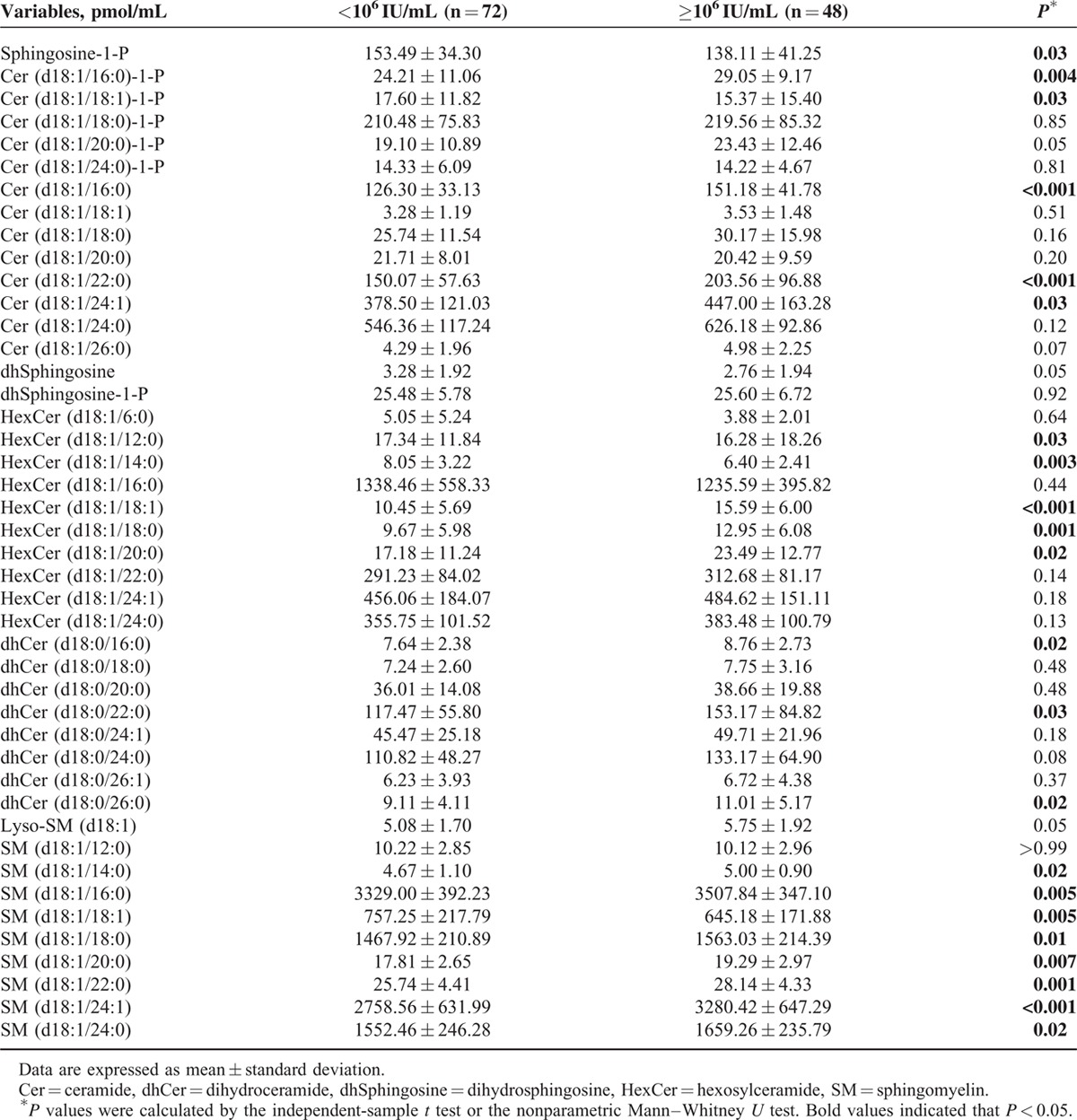
Plasma Sphingolipid Profile of Untreated Chronic Hepatitis C Patients According to HCV-RNA Load

**TABLE 3:**
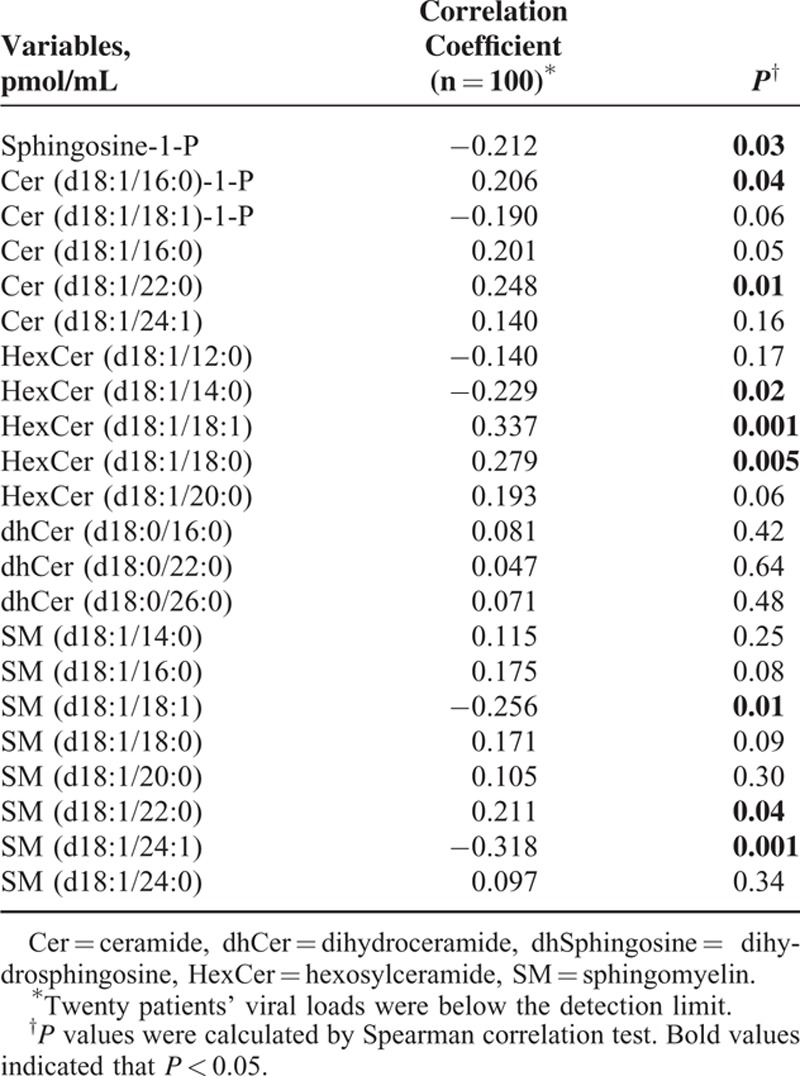
The Correlation Analysis between Differentially Expressed Sphingolipids and HCV-RNA Load in Patients With Chronic Hepatitis C

Routine blood indicators, such as the platelet (PLT), prealbumin (PALB), apolipoprotein B (APOB), and CD4^+^ T-cell ratio, and CD8^+^ T-cell ratio, had significant differences between LVL and HVL patients (*P* < 0.05). Then, PLT, PALB, APOB, CD4^+^ T-cell ratio, CD8^+^ T-cell ratio, sphingosine-1-P, ceramide (Cer) (d18:1/16:0)-1-P, Cer (d18:1/16:0), Cer (d18:1/22:0), hexosylceramide (HexCer) (d18:1/18:1), HexCer (d18:1/18:0), sphingomyelin (SM) (d18:1/18:1), SM (d18:1/22:0), and SM (d18:1/24:1) were initially included in the multivariate analysis. The odds ratio (OR) for HexCer (d18:1/18:1) and HexCer (d18:1/18:0) reached 1.302 (95% confidence interval [CI] 1.129–1.502) and 0.868 (95% CI 0.757–0.995), respectively (Table 4).

**TABLE 4:**
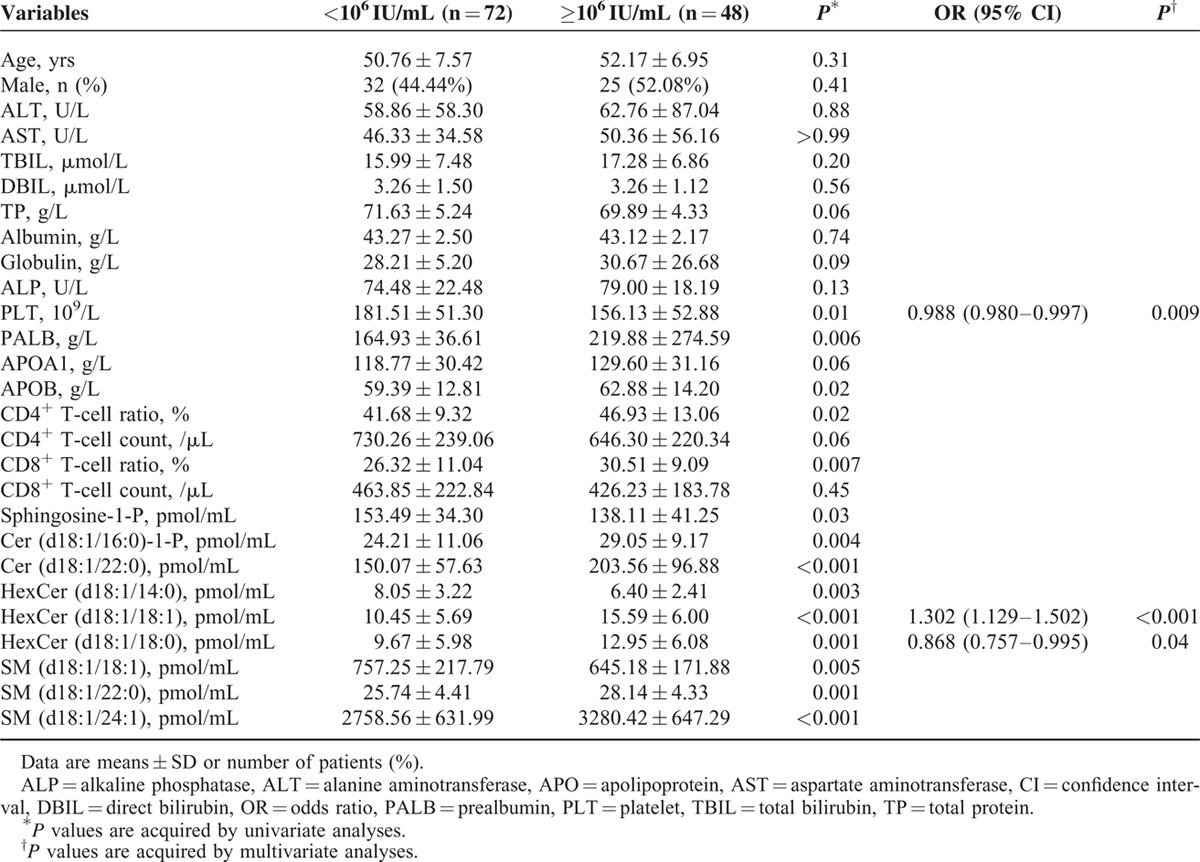
Indicators Related to HCV-RNA Load in Chronic Hepatitis C Patients in Univariate and Multivariate Analyses

### Identification of HCV Replication in the Subgroup of CHC Patients Without Significant Necroinflammation (Grade ≤2)

Hepatic necroinflammatory activity is closely related to the rapid disease progression in CHC patients,^26^ and hepatic inflammation caused by T cells contributes to HCV replication.^27–29^ To reduce the impact of hepatic inflammation and the possible accompanying cytotoxic T-lymphocyte response on HCV replication, we evaluated the association of sphingolipids with the HCV-RNA load in the subgroup of CHC patients without significant necroinflammation (grade ≤2) after adjusting for the immunological index. Routine blood indicators PLT, PALB, complement 3 (C3), and the CD8^+^ T-cell ratio were significantly different between LVL and HVL patients (*P* < 0.05). Cer (d18:1/16:0)-1-P, Cer (d18:1/22:0), HexCer (d18:1/14:0), HexCer (d18:1/18:0), HexCer (d18:1/18:1), Lyso-SM(d18:1), SM (d18:1/18:1), SM (d18:1/20:0), and SM (d18:1/24:1) showed significant differences between LVL and HVL patients (*P* < 0.05) for all plasma sphingolipids (Tables S1 and S2). For the logistic regression analysis, PLT, PALB, C3, CD8^+^ T-cell ratio, Cer (d18:1/16:0)-1-P, Cer (d18:1/22:0), HexCer (d18:1/14:0), HexCer (d18:1/18:0), HexCer (d18:1/18:1), Lyso-SM(d18:1), SM (d18:1/18:1), SM (d18:1/20:0), and SM (d18:1/24:1) were included. The final results showed that the OR for HexCer (d18:1/18:1) reached 1.186 (95% CI 1.085–1.296) (Table 5 ).

**TABLE 5:**
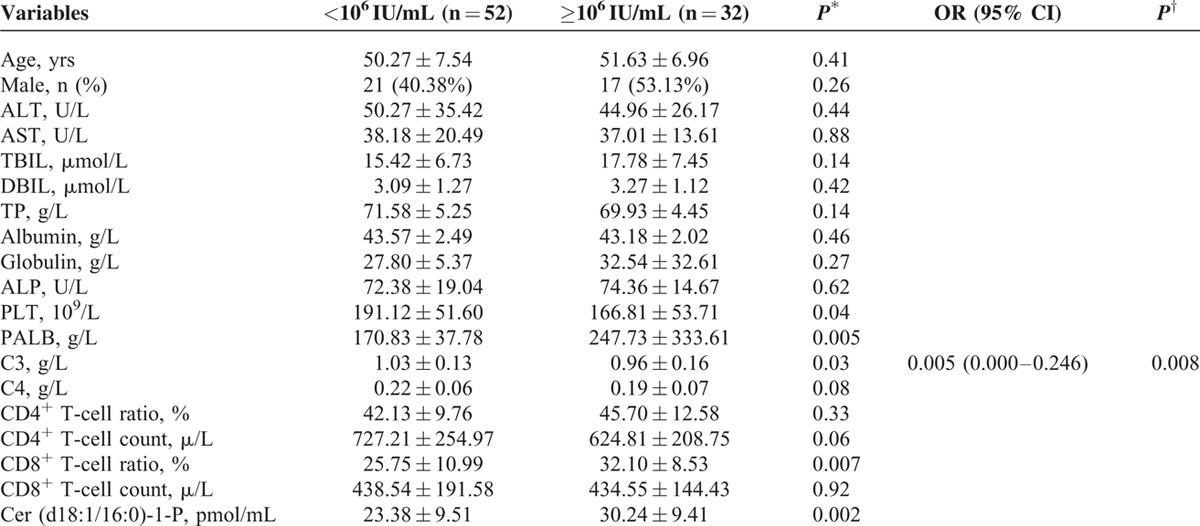
Indicators Related to Sphingolipids in Chronic Hepatitis C Patients With Hepatic Inflammation Grade ≤2 in Univariate and Multivariate Analyses

**TABLE 5 (Continued):**
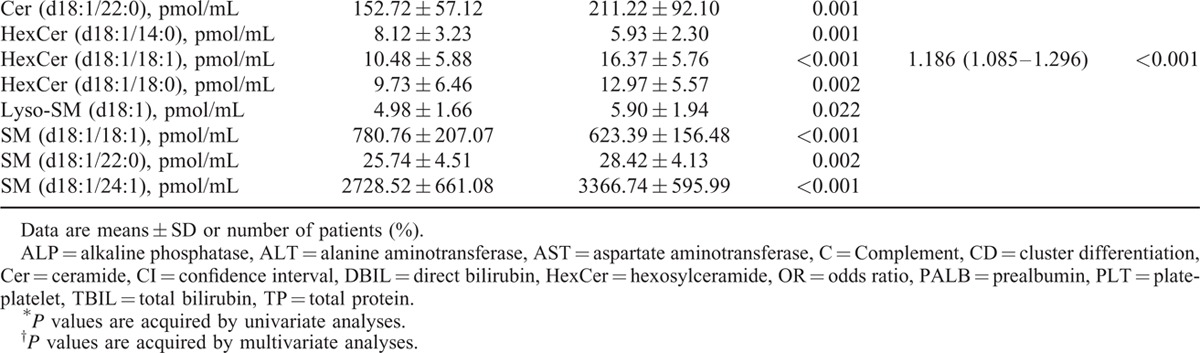
Indicators Related to Sphingolipids in Chronic Hepatitis C Patients With Hepatic Inflammation Grade ≤2 in Univariate and Multivariate Analyses

### Relationship Between Plasma Sphingolipids and HCV-RNA Load in CHC Patients With HCV Genotype 2

The relationship between plasma sphingolipids and the HCV-RNA load in 53 patients with HCV genotype 2 was analyzed. Of all plasma sphingolipids, Cer(d18:1/24:0), HexCer (d18:1/18:1), HexCer (d18:1/18:0), SM (d18:1/18:1), SM (d18:1/22:0), and SM (d18:1/24:1) were significantly different between LVL and HVL patients (*P* < 0.05) in univariate analysis (Tables S3 and S4). Routine blood indicators, such as the total protein (TP), globulin, total bile acid, PALB, APOB, and lgG, showed significant differences between LVL and HVL patients (*P* < 0.05). In plasma sphingolipids, Cer (d18:1/24:0) (OR 1.009, 95% CI 1.001–1.017) and HexCer (d18:1/18:1) (OR 1.173, 95% CI 1.031–1.334) were independently linked to the HCV-RNA load (LVL vs HVL), after adjusting for other factors (Table 6).

**TABLE 6:**
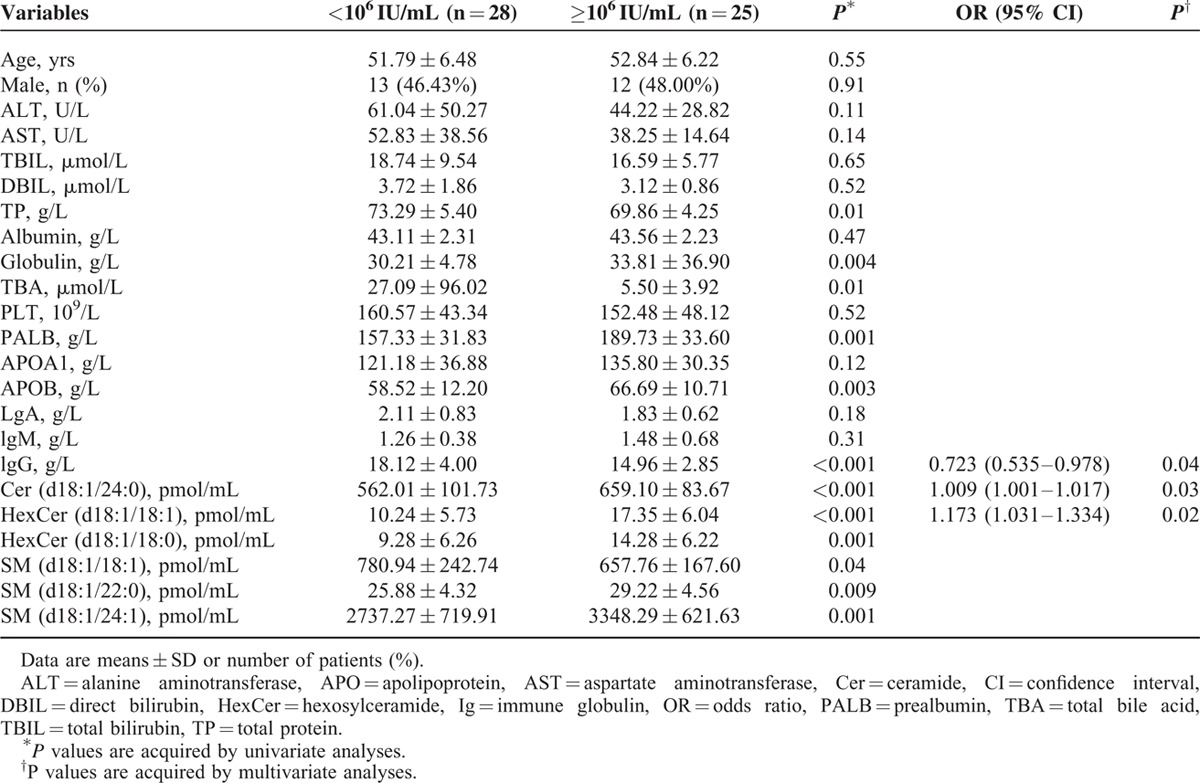
Indicators Related to Sphingolipids in Chronic Hepatitis C Patients With HCV Genotype 2 in Univariate and Multivariate Analyses

## DISCUSSION

In this study, a cohort of antiviral treatment naïve, nondiabetic, and nonobese CHC patients were enrolled. After eliminating the influence of antiviral treatment, we revealed the true relationship between plasma sphingolipids and HCV replication. For the first time, we found that the altered plasma sphingolipids were closely associated with HCV replication in chronic HCV infection, especially for CHC patients with genotype 2.

Sphingolipids have recently been found to be involved in liver disease; for example, they play an important role in hepatitis C virus entry,^9^ HCV infection, virion maturation,^30^ liver fibrosis progression, and antiviral therapy responsiveness.^31^ To the best of our knowledge, the role of plasma sphingolipids in HCV replication of CHC patients is currently unknown. In our study, although several sphingolipids correlate with HCV replication, only HexCer (d18:1/18:1) always performed outstandingly in the subsequent analysis and subgroup analysis.

In the correlation analysis, HexCer (d18:1/18:1) was positively correlated with the HCV-RNA load, which reflects the viral replication level. Additionally, alterations in plasma HexCer (d18:1/18:1) were independently related to HVL after adjusting for confounding variables. Moreover, an in vitro study reported that glycosphingolipids play a crucial role in the HCV life cycle and that they share a link with phosphatidylinositol 4-phosphate in HCV replication.^32^ However, the detailed underlying mechanisms responsible for the association of HexCer (d18:1/18:1) with HCV replication is unclear.

Our previous studies indicated that plasma sphingolipids are related to hepatic inflammation in CHC, especially hepatic necroinflammation (grade >2).^18,19^ To eliminate the impact of necroinflammation on plasma sphingolipids and the possibly accompanying cytotoxic T-lymphocyte response on HCV replication, we analyzed the relationship between plasma sphingolipids and HCV replication in CHC patients without hepatic necroinflammation (grade ≤2). Remarkably, the plasma HexCer (d18:1/18:1) level was also independently related to HCV replication in this subgroup analysis. However, another sphingolipid, HexCer (d18:1/18:0), as an independent factor for HCV replication in CHC patients, was not associated with viral replication in CHC patients who had hepatic inflammation grade ≤2.

In our cohort, HCV genotypes 1b and 2 were the major types, which were consistent with the HCV genotype prevalent in China, where genotypes 1b and 2 account for nearly 80% of HCV infection patients.^33,34^ In CHC patients with genotype 2, the plasma HexCer (d18:1/18:1) level was also independently related to HCV replication after adjusting for the confounding factor in the multivariate analysis. However, Cer (d18:1/24:0), which was an independent factor for viral replication in genotype 2 CHC patients, was not associated with HCV replication in any of the 120 CHC cohort patients. We also explored the role of sphingolipids in CHC patients with genotype 1b. Regrettably, no association was found between these sphingolipids and HCV replication in CHC patients with genotype 1b (data not shown).

Although our study did not reveal the mechanisms for plasma HexCer (d18:1/18:1) in HCV replication on the basis of the present data, we speculated that elevated HexCer (d18:1/18:1) might contribute to the pathogenesis of viral replication in CHC patients, especially for genotype 2 CHC patients. The sample size in this cohort was not sufficiently large, and patients with other genotypes were not analyzed in this study. Therefore, it would make sense to verify the role of HexCer (d18:1/18:1) in large-scale clinical studies in the future.

In conclusion, our results suggest, for the first time, the crucial role of plasma HexCer (d18:1/18:1) in HCV replication. Additionally, HexCer has potential as a novel therapeutic target for antiviral therapy in CHC, especially in patients with genotype 2. These findings will provide new insights into the molecular mechanism of HCV replication in CHC. Further studies are necessary to determine the detailed mechanisms responsible for these associations.

## Supplementary Material

Supplemental Digital Content

## References

[1] Mohd Hanafiah K Groeger J Flaxman AD Global epidemiology of hepatitis C virus infection: new estimates of age-specific antibody to HCV seroprevalence. *Hepatology* 2013; 57:1333–1342. 23172780 10.1002/hep.26141

[2] Hajarizadeh B Grebely J Dore GJ Epidemiology and natural history of HCV infection. *Nature Rev Gastroenterol Hepatol* 2013; 10:553–562. 23817321 10.1038/nrgastro.2013.107

[3] Westbrook RH Dusheiko G Natural history of hepatitis C. *J Hepatol* 2014; 61 (1 Suppl):S58–S68. 25443346 10.1016/j.jhep.2014.07.012

[4] Almasio PL Venezia G Craxi A The impact of antiviral therapy on the course of chronic HCV infection. A systematic review. *Panminerva Medica* 2003; 45:175–182. 14618115

[5] van der Meer-Janssen YP van Galen J Batenburg JJ Lipids in host-pathogen interactions: pathogens exploit the complexity of the host cell lipidome. *Prog Lipid Res* 2010; 49:1–26. 19638285 10.1016/j.plipres.2009.07.003 PMC7112618

[6] Negro F Abnormalities of lipid metabolism in hepatitis C virus infection. *Gut* 2010; 59:1279–1287. 20660700 10.1136/gut.2009.192732

[7] Aizaki H Lee KJ Sung VM Characterization of the hepatitis C virus RNA replication complex associated with lipid rafts. *Virology* 2004; 324:450–461. 15207630 10.1016/j.virol.2004.03.034

[8] Mari M Fernandez-Checa JC Sphingolipid signalling and liver diseases. *Liver Int* 2007; 27:440–450. 17403183 10.1111/j.1478-3231.2007.01475.x

[9] Voisset C Lavie M Helle F Ceramide enrichment of the plasma membrane induces CD81 internalization and inhibits hepatitis C virus entry. *Cell Microbiol* 2008; 10:606–617. 17979982 10.1111/j.1462-5822.2007.01070.x

[10] Umehara T Sudoh M Yasui F Serine palmitoyltransferase inhibitor suppresses HCV replication in a mouse model. *Biochem Biophys Res Commun* 2006; 346:67–73. 16750511 10.1016/j.bbrc.2006.05.085

[11] Sakamoto H Okamoto K Aoki M Host sphingolipid biosynthesis as a target for hepatitis C virus therapy. *Nature Chem Biol* 2005; 1:333–337. 16408072 10.1038/nchembio742

[12] Katsume A Tokunaga Y Hirata Y A serine palmitoyltransferase inhibitor blocks hepatitis C virus replication in human hepatocytes. *Gastroenterology* 2013; 145:865–873. 23791700 10.1053/j.gastro.2013.06.012

[13] Kapadia SB Chisari FV Hepatitis C virus RNA replication is regulated by host geranylgeranylation and fatty acids. *Proc Natl Acad Sci USA* 2005; 102:2561–2566. 15699349 10.1073/pnas.0409834102 PMC549027

[14] Su AI Pezacki JP Wodicka L Genomic analysis of the host response to hepatitis C virus infection. *Proc Natl Acad Sci USA* 2002; 99:15669–15674. 12441396 10.1073/pnas.202608199 PMC137774

[15] Takano T Tsukiyama-Kohara K Hayashi M Augmentation of DHCR24 expression by hepatitis C virus infection facilitates viral replication in hepatocytes. *J Hepatol* 2011; 55:512–521. 21184787 10.1016/j.jhep.2010.12.011

[16] Hirata Y Ikeda K Sudoh M Self-enhancement of hepatitis C virus replication by promotion of specific sphingolipid biosynthesis. *PLoS Pathog* 2012; 8:e1002860. 22916015 10.1371/journal.ppat.1002860 PMC3420934

[17] Qu F Wu CS Hou JF Sphingolipids as new biomarkers for assessment of delayed-type hypersensitivity and response to triptolide. *PloS One* 2012; 7:e52454. 23300675 10.1371/journal.pone.0052454 PMC3530451

[18] Li JF Qu F Zheng SJ Plasma sphingolipids as potential indicators of hepatic necroinflammation in patients with chronic hepatitis C and normal alanine aminotransferase level. *PLoS One* 2014; 9:e95095. 24736528 10.1371/journal.pone.0095095 PMC3988168

[19] Qu F Zheng SJ Wu CS Lipidomic profiling of plasma in patients with chronic hepatitis C infection. *Anal Bioanal Chem* 2014; 406:555–564. 24253410 10.1007/s00216-013-7479-8

[20] Li JF Qu F Zheng SJ Elevated plasma sphingomyelin (d18:1/22:0) is closely related to hepatic steatosis in patients with chronic hepatitis C virus infection. *Eur J Clin Microbiol Infect Dis* 2014; 33:1725–1732. 24810965 10.1007/s10096-014-2123-x

[21] Li JF Qu F Zheng SJ Plasma sphingolipids: potential biomarkers for severe hepatic fibrosis in chronic hepatitis C. *Molec Med Rep* 2015; 12:323–330. 25695872 10.3892/mmr.2015.3361

[22] Li JF Liu S Ren F Fibrosis progression in interferon treatment-naive Chinese plasma donors with chronic hepatitis C for 20 years: a cohort study. *Int J Infect Dis* 2014; 27:49–53. 25168642 10.1016/j.ijid.2014.07.003

[23] Ghany MG Strader DB Thomas DL American Association for the Study of Liver D. Diagnosis, management, and treatment of hepatitis C: an update. *Hepatology* 2009; 49:1335–1374. 19330875 10.1002/hep.22759 PMC7477893

[24] Hepatotogy Branch I Parasitology branch CMA. [Guideline of prevention and treatment of hepatitis C]. *Chin J Prevent Med* 2004; 38:210–215. 15182496

[25] Scheuer PJ Classification of chronic viral hepatitis: a need for reassessment. *J Hepatol* 1991; 13:372–374. 1808228 10.1016/0168-8278(91)90084-o

[26] Ghany MG Kleiner DE Alter H Progression of fibrosis in chronic hepatitis C. *Gastroenterology* 2003; 124:97–104. 12512034 10.1053/gast.2003.50018

[27] Helbig KJ Ruszkiewicz A Semendric L Expression of the CXCR3 ligand I-TAC by hepatocytes in chronic hepatitis C and its correlation with hepatic inflammation. *Hepatology* 2004; 39:1220–1229. 15122750 10.1002/hep.20167

[28] He XS Greenberg HB CD8+ T-cell response against hepatitis C virus. *Viral Immunol* 2002; 15:121–131. 11952134 10.1089/088282402317340279

[29] Freeman AJ Pan Y Harvey CE The presence of an intrahepatic cytotoxic T lymphocyte response is associated with low viral load in patients with chronic hepatitis C virus infection. *J Hepatol* 2003; 38:349–356. 12586302 10.1016/s0168-8278(02)00424-5

[30] Suzuki T Masaki T Aizaki H [Involvement of nonstructural protein 5A and lipids on production of hepatitis C virus particles]. *Uirusu* 2008; 58:199–205. 19374198 10.2222/jsv.58.199

[31] Georgios Grammatikos NF Dimitra B Stephanie S Variations in serum sphingolipid levels associate with liver fibrosis progression and poor treatment outcome in hepatitis C virus but not hepatitis B virus infection. *Hepatology* 2015; 61:812–822. 25348752 10.1002/hep.27587

[32] Khan I Katikaneni DS Han Q Modulation of hepatitis C virus genome replication by glycosphingolipids and four-phosphate adaptor protein 2. *J Virol* 2014; 88:12276–12295. 25122779 10.1128/JVI.00970-14 PMC4248901

[33] Cui Y Jia J Update on epidemiology of hepatitis B and C in China. *J Gastroenterol Hepatol* 2013; 28 Suppl 1:7–10. 23855289 10.1111/jgh.12220

[34] Rao H Wei L Lopez-Talavera JC Distribution and clinical correlates of viral and host genotypes in Chinese patients with chronic hepatitis C virus infection. *J Gastroenterol Hepatol* 2014; 29:545–553. 24090188 10.1111/jgh.12398 PMC4272577

