# Supplementary material for: Up-regulation of Plasma Hexosylceramide (d18:1/18:1) Contributes to Genotype 2 Virus Replication in Chronic Hepatitis C: A 20-Year Cohort Study
Source: Medicine (Baltimore). 2016 Jun 10;95(23):e3773. doi: 10.1097/MD.0000000000003773 (PMC4907656; doi:10.1097/MD.0000000000003773)
Supplement: Supplemental Digital Content [file medi-95-e3773-s001.doc]

| TABLE S1 Plasma Sphingolipid Profile of Untreated Chronic Hepatitis C Patients with Hepatic Inflammation Grade ≤ 2. | | | |
| --- | --- | --- | --- |
| Variable (pmol/ml) | <10^6^ IU/ml  (n=52) | ≥10^6^ IU/ml  (n=32) | P value* |
| Sphingosine-1-P | 152.71±34.04 | 132.62±38.21 | **0.01** |
| Cer(d18:1/16:0)-1-P | 23.38±9.51 | 30.24±9.41 | **0.002** |
| Cer(d18:1/18:1)-1-P | 17.35±11.10 | 14.55±16.20 | **0.01** |
| Cer(d18:1/18:0)-1-P | 209.34±66.90 | 220.93±91.89 | 0.94 |
| Cer(d18:1/20:0)-1-P | 18.35±10.28 | 24.22±12.96 | **0.03** |
| Cer(d18:1/24:0)-1-P | 13.89±5.75 | 14.22±4.21 | 0.78 |
| Cer(d18:1/16:0) | 128.81±34.94 | 150.73±43.69 | **0.01** |
| Cer(d18:1/18:1) | 3.46±1.28 | 3.59±1.56 | 0.92 |
| Cer(d18:1/18:0) | 27.12±12.88 | 29.61±16.61 | 0.59 |
| Cer(d18:1/20:0) | 22.54±8.14 | 19.24±8.45 | **0.04** |
| Cer(d18:1/22:0) | 152.72±57.12 | 211.22±92.10 | **0.001** |
| Cer(d18:1/24:1) | 395.15±127.72 | 452.08±179.58 | 0.22 |
| Cer(d18:1/24:0) | 555.65±117.16 | 629.82±93.31 | **0.003** |
| Cer(d18:1/26:0) | 4.44±2.13 | 5.04±2.39 | 0.17 |
| dhSphingosine | 3.48±2.11 | 2.61±1.73 | **0.04** |
| dhSphingosine-1-P | 25.38±5.99 | 25.16±7.12 | 0.88 |
| HexCer(d18:1/6:0) | 5.82±5.91 | 4.06±1.96 | 0.90 |
| HexCer(d18:1/12:0) | 17.13±11.54 | 14.29±17.56 | **0.01** |
| HexCer(d18:1/14:0) | 8.12±3.23 | 5.93±2.30 | **0.001** |
| HexCer(d18:1/16:0) | 1338.70±512.96 | 1166.97±378.26 | 0.15 |
| HexCer(d18:1/18:1) | 10.48±5.88 | 16.37±5.76 | **<0.001** |
| HexCer(d18:1/18:0) | 9.73±6.46 | 12.97±5.57 | **0.002** |
| HexCer(d18:1/20:0) | 17.22±11.76 | 23.23±12.20 | 0.08 |
| HexCer(d18:1/22:0) | 288.79±86.52 | 300.76±73.16 | 0.45 |
| HexCer(d18:1/24:1) | 449.02±184.25 | 475.63±153.49 | 0.50 |
| HexCer(d18:1/24:0) | 353.01±102.46 | 372.18±94.94 | 0.39 |
| dhCer(d18:0/16:0) | 7.70±2.39 | 8.73±2.88 | 0.12 |
| dhCer(d18:0/18:0) | 7.35±2.59 | 7.44±3.13 | 0.96 |
| dhCer(d18:0/20:0) | 36.59±15.32 | 37.57±18.59 | 0.72 |
| dhCer(d18:0/22:0) | 119.16±61.31 | 156.72±89.65 | 0.08 |
| dhCer(d18:0/24:1) | 43.76±21.26 | 49.20±19.95 | 0.19 |
| dhCer(d18:0/24:0) | 108.71±46.02 | 134.85±69.80 | 0.13 |
| dhCer(d18:0/26:1) | 6.40±4.35 | 6.58±5.08 | 0.98 |
| dhCer(d18:0/26:0) | 9.17±4.50 | 11.35±5.46 | **0.03** |
| Lyso-SM(d18:1) | 4.98±1.66 | 5.90±1.94 | **0.02** |
| SM(d18:1/12:0) | 10.06±2.89 | 9.81±2.89 | 0.910 |
| SM(d18:1/14:0) | 4.60±0.99 | 4.97±0.96 | 0.10 |
| SM(d18:1/16:0) | 3321.68±416.72 | 3534.43±348.72 | **0.007** |
| SM(d18:1/18:1) | 780.76±207.07 | 623.39±156.48 | **<0.001** |
| SM(d18:1/18:0) | 1477.56±225.68 | 1557.64±220.55 | 0.12 |
| SM(d18:1/20:0) | 17.90±2.66 | 19.42±3.07 | **0.02** |
| SM(d18:1/22:0) | 25.74±4.51 | 28.42±4.13 | **0.002** |
| SM(d18:1/24:1) | 2728.52±661.08 | 3366.74±595.99 | **<0.001** |
| SM(d18:1/24:0) | 1555.24±246.95 | 1685.35±232.70 | **0.02** |
| Data are expressed as mean ± standard deviation, P-values* were calculated by Independent-Sample T test tor the nonparametric Mann-Whitney U test.  Cer = ceramide, dhSphingosine = dihydrosphingosine, HexCer = hexosylceramide, dhCer = dihydroceramide, SM = sphingomyelin. | | | |

| TABLE S2 The Correlation Analysis in Plasma Sphingolipids with Significant Difference by HCV Virus Load in Chronic Hepatitis C Patients with Hepatic Inflammation Grade ≤ 2. | | |
| --- | --- | --- |
| Variable (pmol/mL) | Correlation  Coefficient(n=65)* | P value** |
| Sphingosine-1-P | -0.224 | 0.07 |
| Cer(d18:1/16:0)-1-P | 0.294 | **0.02** |
| Cer(d18:1/18:1)-1-P | -0.202 | 0.11 |
| Cer(d18:1/16:0) | 0.140 | 0.27 |
| Cer(d18:1/20:0) | -0.197 | 0.12 |
| Cer(d18:1/22:0) | 0.285 | **0.02** |
| Cer(d18:1/24:0) | 0.231 | 0.06 |
| dhSphingosine | -0.223 | 0.07 |
| HexCer(d18:1/12:0) | -0.198 | 0.11 |
| HexCer(d18:1/14:0) | -0.295 | 0.02 |
| HexCer(d18:1/18:1) | 0.363 | **0.003** |
| HexCer(d18:1/18:0) | 0.301 | **0.02** |
| dhCer(d18:0/26:0) | 0.076 | 0.55 |
| Lyso-SM(d18:1) | 0.252 | **0.04** |
| SM(d18:1/16:0) | 0.223 | 0.07 |
| SM(d18:1/18:1) | -0.351 | **0.004** |
| SM(d18:1/20:0) | 0.118 | 0.35 |
| SM(d18:1/22:0) | 0.265 | **0.03** |
| SM(d18:1/24:1) | 0.367 | **0.003** |
| SM(d18:1/24:0) | 0.135 | 0.28 |
| *ninteen patients’ viral loads were below the detection limit;P-values** were calculated by Spearman Correlation test.  Cer = ceramide, dhSphingosine = dihydrosphingosine, HexCer = hexosylceramide, dhCer = dihydroceramide, SM = sphingomyelin. | | |

| TABLE S3 Plasma Sphingolipids Profile of Untreated Chronic Hepatitis C Patients with Genotype 2. | | | |
| --- | --- | --- | --- |
| Variable (pmol/ml) | <10^6^ IU/ml  (n=28) | ≥10^6^ IU/ml  (n=25) | P value* |
| Sphingosine-1-P | 153.11±35.24 | 139.01±36.33 | 0.15 |
| Cer(d18:1/16:0)-1-P | 25.61±13.46 | 30.48±9.94 | 0.14 |
| Cer(d18:1/18:1)-1-P | 16.36±11.36 | 15.50±17.49 | 0.07 |
| Cer(d18:1/18:0)-1-P | 206.38±88.19 | 220.91±95.69 | 0.76 |
| Cer(d18:1/20:0)-1-P | 19.95±11.72 | 24. 81±13.81 | 0.28 |
| Cer(d18:1/24:0)-1-P | 15.08±7.68 | 14.79±4.16 | 0.48 |
| Cer(d18:1/16:0) | 133.71±34.73 | 160.43±41.97 | **0.01** |
| Cer(d18:1/18:1) | 3.78±1.33 | 3.79±1.69 | 0.32 |
| Cer(d18:1/18:0) | 27.22±14.17 | 32.50±17.56 | 0.22 |
| Cer(d18:1/20:0) | 21.19±7.17 | 20.85±9.35 | 0.55 |
| Cer(d18:1/22:0) | 156.64±65.49 | 213.29±102.92 | **0.005** |
| Cer(d18:1/24:1) | 378.72±113.65 | 449.19±175.64 | 0.09 |
| Cer(d18:1/24:0) | 562.01±101.73 | 659.10±83.67 | **<0.001** |
| Cer(d18:1/26:0) | 4.52±1.90 | 5.54±2.53 | 0.10 |
| dhSphingosine | 3.47±2.22 | 2.71±2.12 | 0.07 |
| dhSphingosine-1-P | 25.03±4.69 | 26.39±7.01 | 0.45 |
| HexCer(d18:1/6:0) | 4.99±5.37 | 4.25±2.30 | 0.39 |
| HexCer(d18:1/12:0) | 20.32±16.86 | 17.01±20.51 | 0.19 |
| HexCer(d18:1/14:0) | 8.30±3.42 | 6.70±2.29 | 0.05 |
| HexCer(d18:1/16:0) | 1344.04±645.85 | 1327.09±438.79 | 0.67 |
| HexCer(d18:1/18:1) | 10.24±5.73 | 17.35±6.04 | **<0.001** |
| HexCer(d18:1/18:0) | 9.28±6.26 | 14.28±6.22 | **0.001** |
| HexCer(d18:1/20:0) | 17.58±11.87 | 25.19±12.37 | **0.03** |
| HexCer(d18:1/22:0) | 289.49±85.46 | 330.76±87.86 | 0.09 |
| HexCer(d18:1/24:1) | 456.58±189.94 | 514.40±155.43 | 0.23 |
| HexCer(d18:1/24:0) | 353.71±112.81 | 403.92±98.89 | 0.09 |
| dhCer(d18:0/16:0) | 8.15±2.52 | 9.03±2.66 | 0.25 |
| dhCer(d18:0/18:0) | 7.62±3.34 | 8.07±3.53 | 0.49 |
| dhCer(d18:0/20:0) | 39.04±13.35 | 40.67±22.92 | 0.76 |
| dhCer(d18:0/22:0) | 126.72±51.45 | 165.23±95.96 | 0.22 |
| dhCer(d18:0/24:1) | 50.87±32.08 | 47.75±20.49 | 0.89 |
| dhCer(d18:0/24:0) | 121.47±50.01 | 143.85±74.83 | 0.36 |
| dhCer(d18:0/26:1) | 7.54±5.39 | 7.31±5.53 | 0.92 |
| dhCer(d18:0/26:0) | 9.68±4.29 | 11.61±6.08 | 0.22 |
| Lyso-SM(d18:1) | 5.02±1.36 | 6.11±2.31 | **0.04** |
| SM(d18:1/12:0) | 10.70±2.25 | 10.03±2.94 | 0.76 |
| SM(d18:1/14:0) | 4.72±1.34 | 5.20±0.91 | **0.01** |
| SM(d18:1/16:0) | 3363.37±483.67 | 3538.58±362.33 | 0.15 |
| SM(d18:1/18:1) | 780.94±242.74 | 657.76±167.60 | **0.04** |
| SM(d18:1/18:0) | 1483.36±227.78 | 1593.79±246.37 | 0.05 |
| SM(d18:1/20:0) | 17.95±2.64 | 20.00±3.30 | **0.02** |
| SM(d18:1/22:0) | 25.88±4.32 | 29.22±4.56 | **0.009** |
| SM(d18:1/24:1) | 2737.27±719.91 | 3348.29±621.63 | **0.001** |
| SM(d18:1/24:0) | 1576.88±219.18 | 1734.67±241.57 | **0.02** |
| Data are expressed as mean ± standard deviation, P-values* were calculated by Independent-Sample T test tor the nonparametric Mann-Whitney U test.  Cer = ceramide, dhSphingosine = dihydrosphingosine, HexCer = hexosylceramide, dhCer = dihydroceramide, SM = sphingomyelin. | | | |

| TABLE S4 The Correlation Analysis in Plasma Sphingolipids with Significant Difference by HCV Virus Load in Chronic Hepatitis C Patients with Genotype 2. | | |
| --- | --- | --- |
| Variable (pmol/mL) | Correlation  Coefficient(n=52)* | P value** |
| Cer(d18:1/16:0) | 0.209 | 0.14 |
| Cer(d18:1/22:0) | 0.278 | 0.05 |
| Cer(d18:1/24:0) | 0.344 | **0.01** |
| HexCer(d18:1/18:1) | 0.439 | **0.001** |
| HexCer(d18:1/18:0) | 0.330 | **0.02** |
| HexCer(d18:1/20:0) | 0.194 | 0.17 |
| Lyso-SM(d18:1) | 0.264 | 0.06 |
| SM(d18:1/14:0) | 0.197 | 0.16 |
| SM(d18:1/18:1) | -0.286 | **0.04** |
| SM(d18:1/20:0) | 0.186 | 0.19 |
| SM(d18:1/22:0) | 0.337 | **0.01** |
| SM(d18:1/24:1) | 0.376 | **0.006** |
| SM(d18:1/24:0) | 0.201 | 0.15 |
| *Eleven patients’ viral loads were below the detection limit; P-values** were calculated by Spearman Correlation test.  Cer = ceramide, dhSphingosine = dihydrosphingosine, HexCer = hexosylceramide, dhCer = dihydroceramide, SM = sphingomyelin. | | |
